# Optimizing Surgical Margins in Breast Conservation

**DOI:** 10.1155/2012/585670

**Published:** 2012-12-09

**Authors:** Preya Ananthakrishnan, Fatih Levent Balci, Joseph P. Crowe

**Affiliations:** ^1^Breast Surgery Division, Department of Surgery, Columbia University College of Physicians and Surgeons, New York, NY 10032, USA; ^2^Department of General Surgery, Cleveland Clinic Foundation, Cleveland, OH 44195, USA

## Abstract

Adequate surgical margins in breast-conserving surgery for breast cancer have traditionally been viewed as a predictor of local recurrence rates. There is still no consensus on what constitutes an adequate surgical margin, however it is clear that there is a trade-off between widely clear margins and acceptable cosmesis. Preoperative approaches to plan extent of resection with appropriate margins (in the setting of surgery first as well as after neoadjuvant chemotherapy,) include mammography, US, and MRI. Improvements have been made in preoperative lesion localization strategies for surgery, as well as intraoperative specimen assessment, in order to ensure complete removal of imaging findings and facilitate margin clearance. Intraoperative strategies to accurately assess tumor and cavity margins include cavity shave techniques, as well as novel technologies for margin probes. Ablative techniques, including radiofrequency ablation as well as intraoperative radiation, may be used to extend tumor-free margins without resecting additional tissue. Oncoplastic techniques allow for wider resections while maintaining cosmesis and have acceptable local recurrence rates, however often involve surgery on the contralateral breast. As systemic therapy for breast cancer continues to improve, it is unclear what the importance of surgical margins on local control rates will be in the future.

## 1. Introduction

Breast-conservation therapy (BCT), including lumpectomy and sentinel lymph node biopsy followed by radiation therapy, is the treatment of choice for women with early stage breast cancer. Randomized trials have shown that overall survival of women undergoing BCT is equivalent to mastectomy [1, 2]. The goal of lumpectomy is to completely excise the tumor with negative margins while maintaining acceptable cosmesis. Rates of margin positivity at initial lumpectomy have been reported ranging from 15% to 47% [3–6]. Positive margins are usually addressed with surgical reexcision, since the risk of local recurrence associated with a positive margin is approximately 2 to 3 times that compared with a negative margin [7]. Reexcision can include reoperative lumpectomy or possibly mastectomy. This additional surgical reoperative procedure can result in increased psychological trauma to the patient, delay of adjuvant therapy, worsened cosmesis, and increased cost [7]. 

It is well accepted that complete removal of tumor is necessary, however, there is aconsiderable debate regarding what margin of normal tissue surrounding the tumor constitutes a negative margin. Definitions range from no ink on tumor surface (NSABP B-06) to 1 cm or more [8]. Blair et al. sent a survey to nearly 1000 breast cancer surgeons, and found that 15% defined a negative margin as no tumor on inked margin, 21% accepted a 1 mm margin, 50% accepted a 2 mm margin, 12% accepted a 5 mm margin, and 3% accepted a 1 cm margin [9]. A meta-analysis by Wang et al. found that wider margins minimize the risk of ipsilateral local recurrence, with lowest recurrence rates achieved with a negative margin larger than 10 mm rather than 2 mm. This finding was independent of whether or not the patient received radiation [10].

In another meta-analysis of 21 retrospective studies which included 14,571 patients, Houssami et al. demonstrated an odds ratio for local recurrence of 2.42 (*P* < 0.001) with positive margins. This meta-analysis did not identify a statistically significant difference in local recurrence associated with margin widths of more than 1 mm, more than 2 mm, or more than 5 mm after adjustment for a radiation boost and endocrine therapy [11]. This suggests that a 2 or 5 mm margin is not necessarily better than a 1 mm margin. 

When considering optimal margin width, it is useful to remember that a “negative” margin does not indicate the absence of residual unresected tumor in the breast [12]. It simply suggests that the residual tumor burden is probably low enough to be controlled with radiotherapy. Even the widest margins resulting from mastectomy do not eliminate risk of local recurrence. This indicates that residual disease burden is not totally eliminated by local surgery and that tumor biology, radiation therapy, and systemic therapy may play an important role in controlling local recurrence [13]. 

In further defining this idea of residual disease burden, Margenthaler et al. have proposed calculating a “margin index” as a predictive tool for residual disease after breast-conservation surgery [14]. This margin index is calculated by dividing the closest margin (in mm) by the tumor size (in mm) ×  100. They found that with a margin index >5, the risk of residual disease was 3.2%. With a margin index of 20, no residual disease was found in the reexcision specimen. 

The NSABP B-06 study showed that in 1851 patients who underwent breast conservation, the positive margin rate was 6.8% and the in-breast tumor recurrence rate was 14.2% over 20 years of followup [1]. Other randomized controlled trials described a range of local recurrences rates from 5.9% at 20 years to 19.7% at 13 years [22]. These randomized trials do not explicitly define margin width, which ranged from no ink on tumor to 1 cm gross margin. While the B-06 trial was conducted in the 1970s, several subsequent NSABP trials in the 1990s showed improvement in 10-year local recurrence rates ranging from 3.5% to 6.5% [23]. Although developments in breast imaging and pathological evaluation of lumpectomy specimens probably contributed to these improvements, significant strides were also made in systemic therapy during this time. This suggests that the likelihood of local recurrence is related to not only the surgical margin width as well, but also to the underlying tumor biology as well as the effectiveness of adjuvant therapy.

Multiple retrospective studies have attempted to define predictors of a positive margin at lumpectomy. These studies identified a number of independent predictors of local recurrence including age less than 40 years, microcalcifications on mammography, palpable tumors, large tumors, multi-centricity, presence of DCIS or lobular histology, and lymphovascular invasion [24]. While these studies showed that 1-2 mm margins were associated with decreased local recurrence rates, it is unclear what the impact of improved systemic therapy and boost radiation therapy is on these results. Cabioglu retrospectively assessed patient and tumor characteristics as well as IBTR rates in two cohorts of patients (those treated from 1970 to 1993, and those treated from 1994 to 1996) [25]. Patients treated after 1994 were less likely to have positive or unknown margin status (2.9% compared to 24.1% before 1994,) and the 5-year IBTR rate was lower in patients treated after 1994 (1.3% compared to 5.7% in those treated before 1994). These investigators postulated that multidisciplinary management, including improvements in pathologic evaluation and systemic therapy, could be credited for the improvement in IBTR. 

Further evidence supports the fact that systemic treatments not only reduce the risk of distant metastases but also reduce the risk of local recurrence. In the NSABP B-14 trial, women with node-negative, estrogen-receptor (ER)-positive tumors were randomly assigned to tamoxifen or placebo [26]. The 10-year rate of local recurrence after breast-conserving surgery was reduced from 14.7% in the placebo group to 4.3% in the tamoxifen group. Similarly, in the NSABP B-13 trial, women with node-negative, ER-negative tumors were randomly assigned to methotrexate and fluorouracil or to no treatment [27]. A reduction was noted in the 10-year local recurrence rate from 13.4% in the no-treatment group to 2.6% in the treatment group. In both studies, the NSABP definition of no ink on tumor was used to define a negative margin.

Studies examining the effect of adding trastuzumab to adjuvant chemotherapy in women with human epidermal growth factor receptor 2 (HER2)-overexpressing tumors have shown an additional 40% reduction in the risk of local recurrence over a median follow-up of 1.5 to 2.0 years [28]. Triple negative tumors have the highest risk of local recurrence after both breast-conserving therapy and mastectomy [29–31], and retrospective studies do not show an improvement in local control after mastectomy as compared with lumpectomy and radiation in this subgroup of patients with biologically aggressive tumors [32, 33].

The effect of tumor biology on local recurrence was clearly shown in a study examining the usefulness of the 21-gene recurrence score (Oncotype DX) in predicting local and regional recurrence [34]. The recurrence score was developed to predict the likelihood of distant metastases in patients with ER-positive, node-negative breast cancer who received tamoxifen [35]. Mamounas et al. found that without systemic therapy, 18.4% of patients with a high recurrence score (≥31) had a recurrence of local or regional disease [34]. The addition of tamoxifen had a minimal effect on the rate of local and regional recurrence, with a decrease to 15.8%. In contrast, the combination of chemotherapy and tamoxifen was associated with a reduction in the local recurrence rate to 7.8%. 

Interestingly, the majority of the studies describing local recurrence rates do not make the distinction between true local recurrences and new ipsilateral primary tumors. Yi et al. suggested that approximately 50% of IBTRs are actually new primary cancers as differentiated by histologic subtype and receptor status [36]. This would lead us to expect that the true local recurrence rate may be half of what is reported in the above studies, if in fact half of in-breast recurrences are new primaries. These new primary tumors therefore would not be expected to be affected by margin width. 

## 2. Preoperative Imaging and Treatment ****Strategies

Thorough preoperative imaging is necessary to plan the extent of resection while minimizing positive margins. Standard preoperative imaging includes mammography and ultrasound, and often MRI. Mammography can delineate tumor size and borders, as well as identify extent of microcalcifications, presence of multifocality, and multicentricity. Mammography is also important for assessment of the contralateral breast. Compared to mammography, ultrasonography can often give more accurate estimation of tumor size and borders, particularly in patients of young age with dense breasts.

 MRI is a more sensitive test that can detect additional foci of disease not appreciated on mammogram and ultrasound. Houssami et al., in a metaanalysis of 19 studies, found that MRI detected additional disease in 16% and led to more extended surgery in 5.5% with a change from lumpectomy to mastectomy in 1.1% [37]. Crowe et al. demonstrated that MRI identified occult or separate tumors in 13% of patients [38]. MRI has a high false-positive rate, so it is clear that additional lesions identified on MRI must be biopsied to demonstrate malignancy prior to changes in surgical planning. Of note, the clinical consequence of detecting these additional lesions on MRI is unknown since no study has demonstrated that use of MRI translates into improved local recurrence rates or survival. 

 Another theoretical advantage of MRI is the potential to better define the extent of the index lesion in order to better plan surgical resection. However, Bleicher et al. in a retrospective review of 577 patients (130 of which had preoperative MRI) failed to demonstrate a difference in margin positivity or the need to convert from breast conservation to mastectomy in the group who had MRI [39]. At this time, preoperative MRI does not improve surgical planning and does not reduce the need for reexcision. Furthermore, Shin et al. in a retrospective analysis showed that breast MRI provided more accurate estimation of tumor size in comparison to ultrasound for both invasive and in situ breast cancer. However, no clear benefit in terms of lower reexcision rate, higher rate of success of breast conservation, or reduced rate of local recurrence emerged with routine use of breast MRI before BCT [40].

There is some suggestion that MRI may be better at assessing DCIS than conventional imaging. Kropcho et al. prospectively evaluated patients diagnosed with DCIS with and without MRI [41]. In this study, the correlation between MRI and tumor size was found to be significantly higher; however, no significant difference was found in between-group analysis of the incidence of margin involvement with MRI versus without MRI (30% versus 24.7%, *P* = 0.414, resp.). 

 Neoadjuvant chemotherapy can often shrink larger tumors to allow for breast conservation. Sweeting et al. demonstrated that over 6-year median followup in young women <age 45, locoregional recurrence rates were no different after breast conservation than mastectomy (13% versus 18%) in patients who underwent neoadjuvant chemotherapy [42]. Higher posttreatment, but not pretreatment, stage was associated with higher locoregional recurrence rates. Recently, Moon et al demonstrated that the accuracy of MRI after neoadjuvant chemotherapy is influenced by the molecular subtype of the tumor. MRI was most accurate in predicting residual tumor extent for triple-negative breast tumors, and least accurate in the Luminal A subtype (Pearson correlation coefficient of 0.754 and 0.531.) 

Multivariate analysis suggested that ER status was an independent factor which influenced the accuracy of MRI. In HER2 amplified tumors, the use of HER2-targeted agents was associated with a less accurate MRI prediction of residual tumor extent.

Huang et al. proposed a prognostic index score for patients receiving neoadjuvant chemotherapy composed of four points: (1) clinical N2 to N3 disease, (2) lymphovascular invasion, (3) pathologic size >2 cm, and (4) multifocal residual disease [43]. Patients with an index of 0 or 1 had similar LRR rates between mastectomy and BCT. Patients with a score of 2 had a trend towards less LRR that was not significant (12% after mastectomy versus 28% after BCT), and patients with a score of 3 or 4 had a significant difference (19% after mastectomy versus 61% after BCT.) This index provides a framework in which to guide surgery selection after neoadjuvant chemotherapy, however, does not explicitly address the impact of margin status on LRR rates. 

Other novel preoperative imaging strategies include optical spectroscopy and molecular vibrational imaging. Optical spectroscopy uses properties of tissue microstructure and biochemical composition to characterize tissue. It can differentiate normal from malignant tissue by distinguishing deoxy-hemoglobin, oxy-hemoglobin, water, and lipids, and thus is not limited by mammographic tissue density. This has also shown promise in assessing tumor response to neoadjuvant chemotherapy [44]. This technology is limited in distinguishing DCIS from normal tissue. Molecular vibrational imaging is another quantitative imaging technology that uses Coherent anti-Stokes Raman scattering (CARS) microscopy to visualize cellular and tissue features. This technology shows promise in differentiating invasive ductal from invasive lobular lesions, as well as DCIS from normal tissue. 

## 3. Lesion Localization, Margin Assessment, and Intraoperative Techniques

Preoperative tumor localization for nonpalpable lesions was traditionally performed by the radiologist with either a mammographically or sonographically guided wire placement into the tumor. The limitation of this technique is that it identifies the lesion in one plane only, with limited ability to guide a three-dimensional resection of the lesion. Lesion bracketing with multiple guidewires as opposed to a single wire would theoretically improve margin clearance by facilitating complete resection of an imaging abnormality. However, Liberman et al. found that while bracketing a lesion (particularly if the lesion was a large area of calcifications) with multiple wires may help to ensure removal of the entire mammographic lesion, it still did not improve on rates of margin positivity [45]. 

 Intraoperative specimen radiography using the Faxitron can be done immediately after specimen excision. The Faxitron allows the surgeon to visualize an eccentric location of a tumor or clip so that additional tissue can be removed. Bathla et al. demonstrated a reexcision rate of 14.3% when 2-dimensional Faxitron was used to guide further tissue removal at the time of initial lumpectomy [46]. In this study, 95.8% of patients who would have required subsequent reexcision were spared further surgery since additional margins were taken at the time of lumpectomy based on Faxitron imaging findings. 

 Intraoperative ultrasonography allows for improved guidance on extent of resection. This technique is quite promising for lesions that can be visualized with ultrasound. This was demonstrated by Rahusen et al. in a randomized clinical study comparing ultrasound guided lumpectomy of nonpalpable breast cancer to wire-guided resection. Using ultrasound to localize the cancer improved rates of margin positivity from 45% with wire guided localization alone to 11% with intraoperative US localization [47]. However, many lesions are not visualized on ultrasound; in particular DCIS lesions which are diagnosed as calcifications on mammography often have no ultrasound correlate. For this reason, it is essential for the surgeon to document presence of the lesion on ultrasound preoperatively to ensure visualization. 

 For lesions not visible on ultrasound, a hydrogel based-breast biopsy clip can be placed at the time of biopsy. This clip is visible on ultrasound and enables the surgeon to use US guidance rather than preoperative wire localization for excision of sonographically occult lesions. However, this approach has limitations. Klein et al. reported that while the clip was very well visualized with intraoperative US, there was a high rate of clip migration either prior to the procedure (6.4%) or when the biopsy cavity was transected (45.2%) [48].

Another technique to enable use of intraoperative ultrasound for lesion excision involves cryoprobe assisted localization (CAL), in which an ultrasound-guided cryoprobe is placed into the tumor to freeze it. This enables the tumor to be easily palpable and visible on ultrasound. Tafra et al. demonstrated that although similar rates of margin positivity (28% with CAL compared to 31% with wire guided localization) and reexcision (19% and 21%) were noted, the cosmetic outcome was improved with CAL since less healthy surrounding tissue around the tumor was removed [49].

 Another technique that is showing promise in improving margin clearance is radioguided occult lesion localization (ROLL). This involves placement of a small radioactive seed under imaging guidance. This seed can be detected with a hand-held gamma probe at the time of surgery. A recent metaanalysis of four randomized controlled trials including 449 patients comparing radioguided seed localization to wire guided localization showed improvement in margin status as well as reoperation rates with the ROLL technique [50]. However, when Krekel et al. compared wire guided localization, intraoperative US localization, and the ROLL technique, the rate of positive margins was the lowest in the intraoperative US group [51]. 

 These studies suggest that the ability to visualize the lesion in multiple dimensions facilitates complete removal, however, rates of margin positivity may still be unchanged. Therefore, efforts have been focused on methods of evaluating the lumpectomy specimen intraoperatively to assess margin positivity. Traditional margin assessment intraoperatively consists of either frozen section histology or imprint cytology. Frozen section histology, while relatively accurate in reflecting margin status, is limited due to time, cost, and loss of tissue for permanent section evaluation. Furthermore this method is very labor intensive and can only examine a limited amount of tissue, with false negative rates reported in 19% of patients [52]. Imprint cytology or “touch prep” involves touching the lumpectomy margins to a glass slide, then fixing and staining them based on the principle that cancer cells will stick to the slide and fat cells will not. This method only assesses tumor cells at the lumpectomy surface and does not indicate when margins are close. The accuracy is extremely variable and experience dependant, with positive predictive values ranging from 21% to 73.6% [53, 54]. In addition, both of these pathologic techniques are limited in their ability to predict invasive lobular cancer as well as DCIS at the margins [52]. 

Besides pathologic techniques to assess margins, significant efforts have been directed towards intraoperative margin probes to assess the lumpectomy specimen margins at the time of surgery. The MarginProbe (TM, Dune Medical Devices) uses radiofrequency spectroscopy to assess margin status. Using this probe, Allweis et al. reported a decrease in reexcision rate from 12.7% to 5.6% [55]. High frequency ultrasound probes have also been developed for intraoperative margin assessment [56]. This technology may have the ability to differentiate carcinomas and precancerous lesions such as ADH from normal tissue. It can also differentiate invasive lobular cancer from normal tissue, which is a limitation of other techniques. 

Dooley et al. described ductoscopy-assisted lumpectomy based on the “sick lobe” hypothesis, with the idea that the entire lobe of the breast containing disease should be evaluated and all affected areas should be removed in order to minimize local recurrence rates [57]. His nonrandomized series showed a lower rate of local failure in those patients who had ductoscopy assisted surgical excision. Furthermore, 42% of patients were noted to have extensive disease within the affected lobe.

Since a primary drawback of large excisions to achieve negative margins is due to removal of excess volume of tissue and resultant cosmetic deformity, several ablative methods have been investigated to provide a larger perimeter of margin clearance without resecting additional tissue. Manenti et al. demonstrated that cryoablation of unifocal small malignant tumors led to complete necrosis in 14 of 15 patients [58]. Laser ablation has been demonstrated to ablate mammographically detected breast cancer [59]. Klimberg et al. have demonstrated that radiofrequency ablation at the time of surgical excision (eRFA) creates a 5–10 mm zone of ablation around the resected tumor, without removing excess of volume of tissue to achieve the same result [60]. These technologies hold promise in achieving wider margins without compromising cosmesis. 

 Since most true in-breast recurrences occur at or near the initial lumpectomy cavity, partial breast intraoperative radiation has been investigated as an alternative to traditional external beam. The use of a single dose of intraoperative radiation using a spherical applicator placed in the surgical cavity was compared to traditional external beam radiation in the TARGIT-A trial [61]. This trial showed that at 4 years of followup in selected patients, a single intraoperative radiation dose is an acceptable alternative to external beam radiotherapy. 

## 4. Pathologic Assessment

There is no universally accepted pathology standard for assessing breast specimens, and translation of intraoperative findings to the pathology lab can be quite difficult. After a lumpectomy specimen is removed from the breast, there may be distortion of the margins due to compression of the specimen for radiographic lesion confirmation. The breast tissue is fatty, and often with compression of the tissue for specimen radiograph to confirm lesion excision, the specimen flattens out or “pancakes,” resulting in distortion of the specimen and spurious positive margins [62]. Furthermore, even with minimal handling, the breast tissue is fatty and often slides off a tumor which remains firm. 

 Therefore, in addition to assessing the lumpectomy specimen margins, surgeons often submit additional tissue from the cavity margins once the primary specimen has been removed (cavity shave margins). Assessing the cavity margins rather than lumpectomy margins is likely a better indicator of presence of residual disease in the cavity since it avoids the issues of compression and specimen processing artifact. The technique involves resecting thin samples of tissue from all 6 margins (superior, inferior, medial, lateral, anterior, and posterior) for pathology evaluation. This technique can direct the surgeon to the exact location of a positive margin in the event that reexcision is necessary; however, the drawback is that it further increases resection volume [63]. Although the volume of tissue resected is increased, Rizzo et al. demonstrated a higher rate of pathologic margin negativity and therefore a lower rate of reoperation with this technique [64]. While there is a cost savings associated with fewer reoperations, there is additional time required by pathology to assess the extra tissue removed and may adversely impact cosmesis. 

 Another challenge as the lumpectomy specimen moves from the operating room to the pathology lab is specimen orientation. Marking sutures have traditionally been placed on 2 or more of the 6 surfaces of a lumpectomy specimen by the surgeon in the operating room, followed by inking of all 6 margins done by the pathologist in the lab. Molina et al. demonstrated that with 2 marking sutures placed by the surgeon, there was a 20% rate of discordance between surgeon and pathologist interpretation of the margins in specimens larger than 20 square cm [65]. In smaller specimens less than 20 square cm, the discordance was as high as 78%. 

 Particularly disturbing for the surgeon are cases where a positive margin is noted on pathology from the initial lumpectomy, and no further disease is evident on reexcision, since it is unclear whether the reexcision removed the correct area. Dooley and Parker demonstrated that when a single margin was close or positive, reexcision showed tumor in only 35% of cases [66]. When multiple margins were close or positive, reexcision showed tumor in 47% of cases. 

 Pathologic processing includes inking with close attention so that ink does not run into cut surfaces. Multiple samples are taken perpendicular to each inked surface, with additional samples taken based on gross appearance of the tissue [67]. In order to more accurately orient the specimen for the pathologist and to help guide reexcision, Singh et al. compared standard inking by the pathologist after lumpectomy versus intraoperative inking with surgeon input [68]. This study demonstrated a decrease in margin positivity rate from 46% to 23%, as well as a decrease in reexcision rates from 38% to 19% when the surgeon was responsible for inking the margin. Importantly, residual disease at the time of reexcision was noted to be 67% in the group inked by the surgeon (as opposed to 23% in the group inked by the pathologist). This simple technique of surgeon staining the lumpectomy specimen with 6 different ink colors at the time of lumpectomy can enable orientation to be maintained when evaluating the margins. Furthermore, directed reexcision also decreases the volume of tissue excised when compared to the whole cavity reexcision [69]. 

## 5. Oncoplastic Surgery to Achieve Wider ****Margins

 Oncoplastic breast surgery combines the principles of cancer resection with plastic surgery to achieve wide tumor-free margins in such a manner as to maximize resection volume while optimizing cosmetic outcome. The two main techniques used involve volume displacement and volume replacement. Volume displacement techniques combine resection with a variety of different breast-reshaping and breast-reduction techniques and include radial ellipse segmentectomy and circumareolar approach. Lesions in the upper or central breast can be resected with the crescent mastopexy, batwing incision, donut mastopexy, and central quadrantectomy. Lesions of the lower breast can be resected with the triangle incision, inframammary incision, and reduction mastopexy [70]. 

 These procedures can be done by the breast surgeon and/or plastic surgeon at the time of cancer resection. Of note, the three dimensional orientation of the tumor bed is frequently altered with these techniques so that identification of the initial resection cavity for postoperative radiation therapy is not possible. At the very least, placement of surgical clips after tumor resection and before oncoplastic reconstruction may be the most accurate method to localize the RT local boost field. Additionally, oncoplastic techniques commonly prevent a simple further excision in the event of positive margins, so that most patients with involved margins will need a mastectomy [71]. Oncoplastic procedures for cancer often result in the need for a contralateral symmetry procedure. The contralateral procedure can be done at the same time as the cancer resection, or at a later time. 

Volume replacement techniques are performed less frequently, and involve autologous tissue flap placement when there is insufficient tissue for a satisfactory cosmetic result. These procedures can retain the volume and shape of the breast and avoid contralateral breast surgery. However, these techniques are more complex, require a donor site, and lead to increased recovery time following autologous tissue harvesting. Autologous flaps for volume replacement include transverse rectus abdominus (TRAM), adipofascial flap, a lateral thoracodorsal flap, a thoracoepigastric flap, an intercostal artery perforator (ICAP) flap, a thoracodorsal artery perforator (TDAP) flap, and a latissimus dorsi (LD) myocutaneous flap [72]. 

Oncoplastic breast conserving surgery (oBCS) has the potential to improve the aesthetic outcome of BCS as well as extending the role of BCS in situations previously considered unsuitable for conservation (large tumors relative to breast size, central and lower pole tumor location, or multifocality). While tumor size, or more precisely tumor-to-breast volume, is a key indication for oBCS, tumor location is an equally important consideration. However, the application of aesthetic techniques for therapeutic purposes must never compromise the main objective of breast cancer surgery: clear margins with good local disease control [72].

 There is now agrowing evidence through prospective series that oncoplastic techniques offer patients a safe oncological outcome (Table 1). Clough et al. from Institute Curie published their first evaluation of 101 patients and concluded that oncoplastic techniques allow larger resections, however a recurrence rate of 9% was reported with median followup of 5 years [15]. Kaur et al. found that a larger volume excision is possible in a subset of patients treated by oncoplastic techniques however; this series reported a re-excision rate of 16% [16]. Giacalone et al. concluded in their study on 74 patients comparing oncoplastic surgery with quadrantectomy that oBCS extends the indications for breast conserving surgery [73]. Asgeirsson et al. from the European Institute of Oncology have reported long-term results with a 5-year local recurrence rate of 3% [74]. A recent Institute Curie review of 540 oncoplastic conservation procedures between 1986 and 2008 revealed a local recurrence rate of 6.8%: they also noted involved or close margins in 18.9% with 9.4% requiring further surgery as a mastectomy [20]. It is possible that oBCS using reduction mammoplasty techniques may be oncologically superior to sBCS by allowing larger excision volumes and wider margins without compromising cosmesis [18, 19, 21, 74, 75].

It appears that oncoplastic breast surgery extends the indications of breast conservation and allows for achievement of large resection volumes with good cosmesis. However, drawbacks include frequent necessity to operate on the contralateral healthy breast, increased cost, and increased possibility of complications delaying adjuvant therapy. While there has been some concern that oncoplastic surgery could confound subsequent mammographic imaging, Roberts et al. demonstrated that in patients who underwent reduction mammoplasty, no increase in subsequent imaging or diagnostic interventions was noted [76]. 

## 6. Looking Forward

 Trends in breast cancer care continue to progress towards less invasive surgical treatment. Recent data from the ACOSOG Z11 trial suggests that axillary dissection may not be of benefit in node positive patients who receive maximal systemic therapy and radiation. As systemic therapy improves, and individualized and targeted approaches evolve, it is unclear what role surgery will play in achieving local control. Primary ablative therapies may make questions of margins obsolete, in that if a tumor is ablated and resolves on imaging, then surgical excision may not be necessary. 

## Figures and Tables

**Table 1:** Oncoplastic surgery and margin involvement, local recurrence rates, and survival rates.

Author	Year	Number of patients	Weight (g)/volume of specimen	Close/involved margins (reexcision/mastectomy)	Local recurrence rate	Survival rate	Median followup (months)
Clough et al. [15]	2003	101	222		9.4%	95.7%	44
Kaur et al. [16]	2005	30	200	16%			
Rietjens et al. [17]	2007	148	198	2.02%	3%	92.47%	74
Giacalone et al. [18]	2006	31	190	21%			
Meretoja et al. [19]	2010	90		12.2%	0%		26
Fitoussi et al. [20]	2010	540	187.7	18.9%	6.8%	92.6%	49
Chakravorty et al. [21]	2012	146	67 (11–1050)	2.7%	4.3%		28
